# Medical Care Capacity for Influenza Outbreaks, Los Angeles

**DOI:** 10.3201/eid0806.010370

**Published:** 2002-06

**Authors:** Carol A. Glaser, Sabrina Gilliam, William W. Thompson, David E. Dassey, Stephen H. Waterman, Mitchell Saruwatari, Stanley Shapiro, Keiji Fukuda

**Affiliations:** * California Department of Health Services, Richmond, California, USA; † Centers for Disease Control and Prevention, Atlanta, Georgia, USA; ‡ County of Los Angeles, Los Angeles, California, USA; § Kaiser Permanente Southern California, Los Angeles, California, USA

**Keywords:** Influenza, disease outbreaks, crowding, emergency services, hospital, hospital bed capacity, bed occupancy

## Abstract

In December 1997, media reported hospital overcrowding and “the worst [flu epidemic] in the past two decades” in Los Angeles County (LAC). We found that rates of pneumonia and influenza deaths, hospitalizations, and claims were substantially higher for the 1997–98 influenza season than the previous six seasons. Hours of emergency medical services (EMS) diversion (when emergency departments could not receive incoming patients) peaked during the influenza seasons studied; the number of EMS diversion hours per season also increased during the seasons 1993–94 to 1997–98, suggesting a decrease in medical care capacity during influenza seasons. Over the seven influenza seasons studied, the number of licensed beds decreased 12%, while the LAC population increased 5%. Our findings suggest that the capacity of health-care systems to handle patient visits during influenza seasons is diminishing.

In December 1997, television and newspaper media reported that high numbers of patients seeking treatment for respiratory illnesses had overwhelmed the capacity of emergency departments and outpatient facilities in Los Angeles County (LAC). The situation was described as a looming health-care disaster (*1*) and the worst influenza epidemic in the previous 2 decades (*2*,*3*).

Influenza viruses infect all age groups and cause annual or near-annual winter epidemics. The health impact of seasonal epidemics is variable, averaging >20,000 excess deaths (i.e., deaths above an expected baseline of deaths in the absence of influenza) and >110,000 excess hospitalizations per year in the United States (*4*). Severe influenza seasons can result in >40,000 excess deaths and >200,000 excess hospitalizations.

Global pandemics of influenza, which occur when novel influenza viruses emerge, happen unpredictably and less frequently (e.g., 1918–19, 1957–58, and 1968–69 in the 20th century) than seasonal epidemics (*5*). However, the resulting elevation in the number of illnesses and deaths can be much greater than during regular influenza epidemics (*4*,*6*). The Centers for Disease Control and Prevention (CDC) projected that a pandemic similar in impact to the 1957 pandemic, widely considered to be a “medium” pandemic, might result in approximately 300,000 to 750,000 excess hospitalizations and 18 million to 42 million excess outpatient visits in the United States (*7*).

(In December 1997, the LAC Acute Communicable Disease Unit, the California Department of Health Services, and CDC conducted a preliminary investigation of situations in which patients were diverted from one emergency facility to another in LAC. Because findings suggested that approximately 65% of LAC health-care facilities had diverted patients to other hospitals because of overcrowding and concerns about the hospitals’ ability to respond to seasonal and pandemic influenza, we studied the impact of the 1997–98 influenza season on LAC hospitals and emergency services.

## Methods

### Study Population

The entire population of LAC was used as the denominator to estimate death rates from pneumonia and influenza (P&I) and the number of licensed beds per 100,000 persons. Six publicly funded LAC hospitals and six Kaiser Permanente Southern California (KPSC) hospitals were used to study hospitalization patterns. KPSC is a not-for-profit group model health maintenance organization in southern California. Rates of hospitalizations in LAC-funded facilities and KPSC were calculated by using population estimates for persons at or below the poverty level (*8*) and the KPSC enrollee population, respectively. LAC-funded hospitals and KPSC hospitals ranged in size from approximately 100 to 2,000 licensed beds and from approximately 100 to 600 beds, respectively.

### Study Periods

Seven influenza seasons (from 1991 to 1998) were studied. A broad 24-week influenza season was defined as the last 12 weeks of 1 year and the first 12 weeks of the following year. Within each season, we further defined a peak influenza period as the 4-consecutive-week period in which the greatest total number of influenza isolates and antigen detections were reported to the U.S. World Health Organization (WHO) influenza laboratories in Region IX (California, Washington, Oregon, and Hawaii). The peak influenza periods were defined independently of hospitalization, KPSC claims, or emergency medical services (EMS) data.

 P&I death data for LAC were obtained from the California Department of Health Services Vital Statistics Section. A P&I death was defined with a code 480–487, International Classification of Diseases, Ninth Revision (ICD-9). These ICD-9 codes include viral pneumonia, pneumococcal pneumonia, other bacterial pneumonia, pneumonia due to other specified organisms, pneumonia classified elsewhere, bronchopneumonia, pneumonia-organism unspecified, and influenza.

P&I hospitalization data were obtained directly from the six LAC-funded hospitals and KPSC. For LAC-funded hospitals, a P&I hospitalization was defined as one in which one of the first three discharge codes included ICD-9 codes 480–487 (no principal diagnosis was available). For KPSC, a P&I hospitalization was defined as a hospitalization for which the principal discharge diagnosis was assigned an ICD-9 code of 480–487. Hospital data limitations prevented us from using an identical P&I hospitalization definition for both the LAC and KPSC facilities. Hospitalization data included the facility name and the patient's sex, age, discharge date, and disposition at discharge.

Data from claims related to P&I hospitalizations (ICD-9 480–487) were obtained from KPSC**.** A claim is a bill or charge generated by an outside facility when a KPSC patient receives a medical service (e.g., a radiograph or administration of a medication) while hospitalized at a non-KPSC facility. Claims usually are generated when a KPSC facility is diverting patients or when a KPSC patient is too sick to be transported to another KPSC facility. Since a separate claim is generated for each service, one hospitalization usually results in multiple claims. These data, therefore, were analyzed separately from the hospitalization data.

A hospital was considered to be on EMS diversion when its emergency department could not receive incoming patients transported by an Advanced Life Support (ALS) unit. The number of hours that LAC emergency rooms were on diversion for March 1993 to March 1998 was obtained through the LAC Local Emergency Medical Service Agency (LEMSA). Data were not available for periods before March 1993. Under California statute, all ALS diversions require LEMSA approval. LEMSA may deny such requests during periods when regional patient volume is high and extended transport time may have a negative health effect on patients (*9*). This study analyzed only EMS diversions because of emergency department saturation.

We obtained the total number of general acute-care facilities in LAC and licensed beds in each facility, for 1991 to 1997, from data compiled by the Office of Statewide Health Planning and Development, California Department of Health Services (unpub. data). Licensed beds are those licensed to a particular facility regardless of availability for patient care; the total was obtained from the number of beds that appeared on each facility’s license on the last day of the calendar year. Staffed beds are those available for patient care based on current staffing levels. Reliable numbers on staffed beds were unavailable.

### Statistical Methods

Rates of P&I deaths, P&I hospitalizations at LAC-funded facilities and KPSC hospitals, and KPSC claims were calculated for each influenza season from 1991–92 to 1997–98. Rates of P&I deaths, hospitalizations, and KPSC claims during the 4-week peak influenza period were compared with the rates for the remaining 20 weeks of the influenza season by the Mantel-Haenszel common odds ratio (OR) in Stat-Xact 3.0 (*10*). In addition, rates of P&I-associated deaths, hospitalizations, and KPSC claims during the peak influenza period of 1997–98 were compared with the rates in the other six peak periods (1991–92 through 1996–97). Person-week denominators were calculated for peak and nonpeak influenza seasons by using LAC population for each given year and the number of weeks in the period studied (for peak period, 4 weeks; for nonpeak periods, 20 weeks).

## Results

In 1997–98, the 4-week period with the most influenza detections was 52–2 (i.e., weeks 52 and 53 in 1997; weeks 1 and 2 in 1998), when a total of 445 viral isolations and positive antigen tests were reported (Table 1). Most of the other peak periods also occurred in late December and early January.

**Table 1:** Peak period of influenza detections^a^ reported to the World Health Organization’s influenza laboratories, U.S. Region IX, 1991–1998

	Influenza season						
	1991–92	1992–93	1993–94	1994–95	1995–96	1996–97	1997–98
Peak no. of influenza detections/ 4-wk period (% positive)	192 (28)	57 (10)	173 (26)	61 (13)	164 (21)	117 (22)	445 (27)
Peak 4-wk period week no.	2–5	53 ^b^–3	52–3	9–12	51–2	51–2	52 ^b^–2

^a^ Includes viral isolations and positive antigen tests. ^b^Year extended over 53 weeks.

During each of the seven influenza seasons studied, rates of P&I deaths were consistently higher in the 4-week peak influenza period than the other 20 weeks of each influenza season (OR 1.57; 95% confidence interval [CI] 1.50 to 1.64; p<0.001) (Table 2). The rate of P&I deaths during the 1997–98 peak influenza period (16.3 deaths per million person-weeks) was significantly higher (p<0.001) than the rates in the other six peak periods (6.9–12.3 deaths per million person-weeks).-

**Table 2:** Rates of pneumonia and influenza (P&I) deaths, hospitalizations, and claims^a^

		P&I deaths			LAC hospitalizationsb			KPSC hospitalizationsc			KPSC claims		
Influenza period by season		Count	Person-wksd	Rate	Count	Person-wksd	Rate	Count	Person-wksd	Rate	Count	Person-wksd	Rate
Peak 4-wk period													
	91–92	345	36	9.5	157	8	20.6	262	5	52.4	32	5	6.4
	92–93	287	37	7.8	172	8	21.7	235	5	48.5	148	5	30.5
	93–94	457	37	12.3	277	9	31.8	355	5	75.2	257	5	54.5
	94–95	257	37	6.9	214	9	23.6	190	5	40.5	239	5	51.0
	95–96	348	37	9.3	246	9	27.4	330	5	67.3	564	5	115.0
	96–97	333	38	8.8	192	9	22.6	308	5	58.1	715	5	134.8
	97–98	624	38	16.3	648	9	75.3	482	6	84.3	1,0	6	265.9
Nonpeak 20-wk period													
	91–92	1,177	182	6.5	570	38	15.0	1,065	25	42.6	78	25	3.1
	92–93	1,044	184	5.7	796	40	20.1	976	24	40.3	542	24	22.4
	93–94	1,252	186	6.7	737	44	16.9	955	24	40.5	618	24	26.2
	94–95	1,081	187	5.8	924	45	20.4	915	23	39.0	947	23	40.4
	95–96	1,126	187	6.0	710	45	15.8	931	25	38.0	1,419	25	57.9
	96–97	1,319	189	7.0	808	43	19.0	882	27	33.3	2,338	27	88.2
	97–98	1,453	192	7.6	1,610	43	37.4	927	29	32.4	2,548	29	89.1

^a^Abbreviations used: LAC, Los Angeles County; KPSC, Kaiser Permanente Southern California. ^b^Represents six LAC-funded hospitals. ^c^Represents six KPSC hospitals. ^d^Represents 1 million person-weeks.

At the LAC-funded facilities, rates of P&I hospitalizations were also consistently higher during the 4-week peak influenza period than in the other 20 weeks in the influenza season (OR 1.47; 95% CI 1.39 to 1.55; p<0.001) (Table 2). The rate of P&I hospitalizations during the 1997–98 peak period (75.3 hospitalizations per million person-weeks) was significantly higher than the rates during the six other peak periods (20.6–31.8 hospitalizations per million person-weeks) (OR 1.62; 95% CI 1.44 to 1.83; p<0.001).

The rates of P&I hospitalizations and claims in the Kaiser facilities during the 4-week peak periods were significantly higher than rates of P&I hospitalizations and claims during the nonpeak periods (OR 1.63; 95% CI 1.55 to 1.71; p<0.001 for P&I hospitalizations; OR 2.05; 95% CI 1.97 to 2.13; p<0.001 for P&I claims) (Table 2).

Similar to LAC-funded facilities, the rate of P&I hospitalizations in the Kaiser facilities during the 1997–98 peak influenza period was higher than the rate of P&I hospitalizations during the previous six peak periods (OR 1.48; 95% CI 1.3 to 1.64; p<0.001 hospitalizations; OR 4.01; 95% CI 3.75 to 4.29; p<0.001 for P&I claims) (Table 2). In the 1997–98 season, the rate was 84 hospitalizations per million person-weeks, compared with 40–75 hospitalizations per million person-weeks for the previous years studied. The number and rate of KPSC claims were also higher during the peak influenza period of 1997–98 (266 claims per million person-weeks) compared with previous years (range 6–135 claims per million person-weeks) (OR 4.0; 95% CI 3.75 to 4.29; p<0.001).

In the 1997–98 peak influenza period**,** the largest number and percentage of P&I hospitalizations occurred in persons >60 years of age (n=491, 45%), followed by persons <5 years of age (n=166, 15%). This general age distribution was observed in all seven peak influenza periods, but the absolute number of patients was greater for the 1997–98 peak period than for any of the other peak periods.

**Figure 1:**
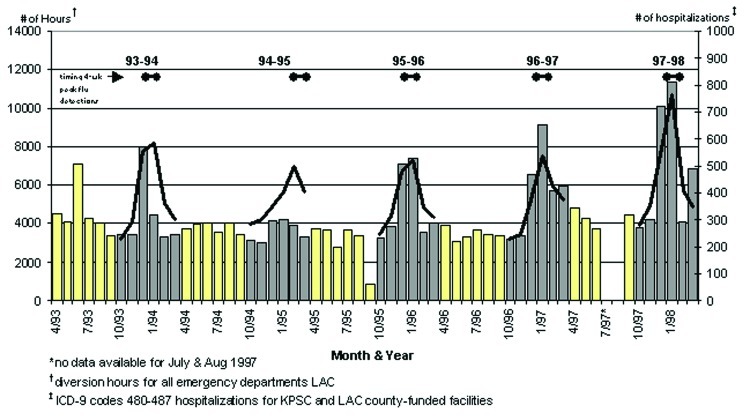
Emergency department diversion hours, influenza hospitalizations, and detection peaks, Los Angeles County, April 1993–March 1998.

The months with the highest number of EMS diversion hours were December 1997 (10,109 hours) and January 1998 (11,388 hours). These months coincided with the peak of the 1997–98 influenza period. From the 1993–94 to the 1997–98 influenza seasons, the number of hours that all LAC hospital emergency departments were on EMS diversion during each December to February (months encompassing all but one of the 4-week influenza peaks) increased from 15,844 hours to 25,584 hours (Figure 1). For comparison, an average of 3,715 EMS diversion hours per month occurred during noninfluenza months in years 1993–1996. Influenza hospitalizations (KPSC and county-funded facilities) and peaks in influenza detections in Figure 1 show the relationship between EMS diversions and influenza activity.

**Figure 2:**
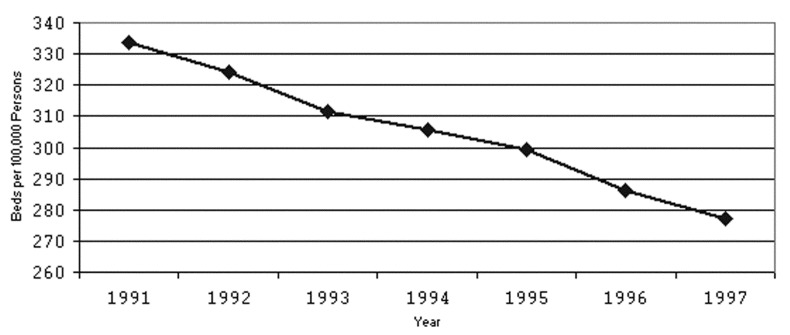
Number of licensed beds per 100,000 persons, Los Angeles County, 1991–1997.

From 1991 to 1997, the number of acute-care hospitals and licensed acute-care hospital beds in LAC decreased from 137 to 130 hospitals and 29,987 to 26,244 licensed beds, respectively. The drop in licensed beds corresponds to a decrease of 334 beds per 100,000 persons to 227 beds per 100,000 persons (Figure 2). Accurate counts of staffed beds were not available.

## Discussion

Several important patterns were observed in this study of LAC hospitalizations and EMS diversion during influenza seasons in LAC from 1991 through 1998. The impact of the influenza season on LAC hospitals was more severe in 1997–98 than in the preceding 6 years. However, although more severe, the elevated levels of hospitalizations and deaths were not unique. In six of seven winters from 1991–92 through 1997–98, similar peaks of P&I hospitalizations and P&I deaths were observed. These peaks correlated with elevated levels of circulating influenza viruses, suggesting that this infection is a key factor leading to increased demands on medical systems in the winter. The study also demonstrated that the number of hours that LAC hospitals were on EMS diversions peaked at approximately the same time that respiratory deaths and hospitalizations peaked. Most importantly, the number of hours that LAC hospitals were on EMS diversion during the peak influenza periods increased substantially over the period of the study. During this period, the LAC population increased, while the number of licensed hospital beds in LAC decreased. If an increasing trend in EMS diversion hours reflects the inability of hospitals to handle critically ill patients, then these contemporaneous patterns call into question the capability of current medical systems to handle regular influenza seasons, as well as more stressful events, such as pandemic influenza.

Our evidence suggests that influenza infections were the major precipitating cause for the annual winter upsurge in patient visits. In this study, we defined the 4-week peak influenza periods on the basis of the number of influenza isolates reported to WHO laboratories in Region IX, not on the basis of hospitalization or death patterns. During these peak influenza periods, levels of respiratory-related deaths, hospitalizations (at both the LAC-funded and KPSC hospitals), and claims for KPSC patients were substantially higher than during the periods when influenza viruses were not in circulation. The patients most frequently hospitalized for P&I-related illnesses (ICD-9 codes 480–487) during the peak influenza weeks were the elderly and the young, a pattern consistent with the epidemiology of influenza. Finally, the number of EMS diversion hours in the 1994–95 season, characterized by light influenza activity, was notably low.

One of the characteristics of influenza epidemics is a highly variable impact on populations. The degree of impact depends on several factors, including prevalence of infections, levels of protective immunity in the population, demographic and health characteristics of the population, and circulating strain. The increased severity of the 1997–98 influenza season was likely due to the appearance of influenza A/Sydney/5/97-like (H3N2) viruses in the United States. This virus, a drift variant of the previously predominant influenza A/Wuhan/359/95 (H3N2) virus strain, first emerged in the spring of 1997 and quickly became the predominant influenza strain in the United States and worldwide (*11*). During the 1997–98 influenza season, this strain accounted for >90% of the influenza virus isolates from Southern California. However, because of the timing of its emergence, this strain was not included in the 1997–98 influenza vaccine (*12*).

The most important observation in our study was the increasing trend in EMS diversion hours during peak influenza periods. One factor in this trend appeared to be a steady erosion in hospital bed capacity despite a growing population in LAC. From 1991 to 1997, the bed capacity in LAC decreased by 17%, when population growth was taken into account. Concomitantly, from 1993–94 through 1997–98, EMS diversion hours combined during the months of December, January, and February increased from 15,844 to 25,584 hours. Since EMS diversion hours reflect times when hospitals are unable to receive critically ill patients, such hours serve as a marker of general overcrowding in emergency rooms and time during which patients are at risk for long waits and poor outcomes (*13*).

Our findings suggest that decreasing bed capacity was an important underlying cause for hospitals’ inability to handle the upsurges in patients and the increasing numbers of hours spent on EMS diversions during the influenza seasons studied. However, we did not examine other potentially important factors, such as nursing shortages, staff illnesses, or limitations in the availability of equipment or intensive-care unit capacity. In a recent study in the emergency rooms in the United States, 14 common causes of emergency room overcrowding were identified, of which 43% were directly related to resource shortages, such as beds and staff (*13*). In a similar survey of emergency department directors in California, 96% of the directors reported overcrowding as a problem and identified increasing numbers of severely ill patients, hospital bed shortages, delays in receiving laboratory results, and nursing shortages as underlying causes (*14*).

These findings have two important implications. First, the near-annual peaking of both P&I hospitalizations and EMS diversions during the influenza season suggests that hospitals and medical systems can and should develop plans to handle the upsurges in patient visits for respiratory illnesses. Second, the increasing number of EMS diversion hours suggests the need to further identify reversible factors responsible for the ongoing erosion in the ability of LAC hospitals to handle upsurges in patient visits.

One important aspect of this study was our decision to restrict the analysis of hospitalizations, KPSC claims, and deaths to ICD9 codes 480–487—codes often used to monitor influenza trends (*15*). In further analyses (not shown), we found that the rates of hospitalizations increased twofold when additional respiratory codes were added and almost threefold when congestive heart failure was included. Other studies have shown that hospitalizations for other respiratory conditions (e.g., bronchitis, chronic airway obstruction) and congestive heart failure increase during influenza season (*16*). These considerations suggest that our analysis of the magnitude of the problem was conservative.

In response to the 1997–98 influenza season, the California Department of Health Services, in collaboration with Kaiser Permanente and the CDC, augmented active influenza surveillance in California. Methods for influenza surveillance were expanded to include monitoring of influenza-related hospitalizations and use of influenza antiviral medications. In addition, surveillance of influenza-like illnesses in outpatient settings and collection of respiratory virus isolation data from several major laboratories throughout the state were also implemented.

Emergency room crowding and diversion is a year-round problem caused by multiple factors within the medical system (*13*,*17*). Our study shows that influenza places an additional stress on an overburdened system. Although we did not study hospitals outside LAC, we think that the situation in LAC is not unique. During the 1997–98 influenza season, media in northern California suggested a similar pattern there (*18*). Furthermore, in January 2000, the New York Times reported emergency rooms were “flooded” secondary to influenza-like illnesses (*19*).

 The ability to handle upsurges in patients is compromised in at least some parts of the country. Action is needed to reverse this situation*.* Because of the 1997–98 influenza season, the Healthcare Association of Southern California, a health-care industry organization, made the following recommendations for hospitals during periods of heavy influenza activity: 1) reduce or eliminate elective surgery; 2) relax staff-versus-patient ratios by working with state licensing agencies; 3) develop methods of identifying and mobilizing additional staff during the winter; 4) establish walk-in influenza clinics to triage and treat patients at lower cost; and 5) develop methods for identifying additional equipment (*20*). This health-care association also recommended influenza immunization programs for staff members and their families early in the season. Implementing such recommendations could alleviate some of the stresses experienced by medical systems in years of increased influenza outbreaks.

Ontario, Canada, implemented a program in 2000–01 that offered influenza vaccines to all its residents to alleviate pressures on its hospitals (*21*). The results of this approach may not be known for several years. The importance of vaccinating persons at high risk for influenza-related complications cannot be overemphasized**.** This step should be widely implemented to reduce hospitalizations and other serious complications of influenza (*22*). Finally, hospitals should also work closely with local and state health departments to obtain up-to-date information about the local circulation of influenza viruses. Such information could be used by hospitals to trigger the implementation of predesignated policies.

## Conclusion

Rebuilding the medical care capacity to handle such patient upsurges will be difficult and expensive. Hospitals in LAC regularly and increasingly exceed their capacity to handle respiratory illness cases. This lack of capacity, along with concerns about the next influenza pandemic and potential terrorist events, suggests that it is time to start this process.
